# Formation and Properties of Biomedical Ti-Ta Foams Prepared from Nanoprecursors by Thermal Dealloying Process

**DOI:** 10.3390/ma12172668

**Published:** 2019-08-22

**Authors:** Grzegorz Adamek, Mikolaj Kozlowski, Mieczyslawa U. Jurczyk, Przemyslaw Wirstlein, Jakub Zurawski, Jaroslaw Jakubowicz

**Affiliations:** 1 Institute of Materials Science and Engineering, Poznan University of Technology, Jana Pawla II 24, 61-138 Poznan, Poland; 2 Division Mother’s and Child’s Health, Poznan University of Medical Sciences, Polna 33, 60-535 Poznan, Poland; 3 Department of Gynecology and Obstetrics, Division of Reproduction, Poznan University of Medical Sciences, Polna 33, 60-535 Poznan, Poland; 4 Department of Immunobiochemistry, Chair of Biology and Environmental Sciences, Poznan University of Medical Sciences, Rokietnicka 8, 60-806 Poznan, Poland

**Keywords:** titanium-based foams, mechanical alloying, thermal dealloying

## Abstract

The paper presents a promising method of preparation of titanium-based foams by the thermal dealloying method. The first step of this study was the Ti-Ta-Mg based nanopowder preparation using the mechanical alloying (MA) process performed at room temperature. The next step was forming the green compacts by cold pressing and then sintering with magnesium dealloying from the titanium-based alloy structure. The mechanism of the porous structure formation was based on the removal of magnesium from the titanium alloy at a temperature higher than the boiling point of magnesium (1090 °C). The influence of the Mg content on the formation of the porous Ti-30Ta foam has been investigated. The sintering stage was performed in vacuum. During the dealloying process, the magnesium atoms diffuse from the middle to the surface of the sample and combine to form vapors and then evaporate leaving pores surrounded by the metallic scaffold. The porosity, the mechanical properties as well as biocompatibility have been investigated. The titanium-based foam of high porosity (up to 76%) and the pore size distribution from nano- to micro-scale have been successfully prepared. For the medical applications, the Ti-Ta metallic foams have shown a positive behavior in the MTT test. The as-shown results clearly exhibit a great potential for thermal dealloying in the preparation of porous structures.

## 1. Introduction

Titanium based materials such as alloys, composites or even pure Ti are the most popular materials for hard tissue implants because of their excellent corrosion resistance, good mechanical properties and superior biocompatibility [1]. Titanium appears in two allotropic forms: Low temperature α and high temperature (above 882 °C) β with hexagonal and cubic crystal structures, respectively. By careful chemical composition and the elimination of toxic elements, it is possible to prepare alloys with excellent biocompatibility and mechanical properties. Alloys with the β structure are the preferred biomaterials because of a much lower Young’s modulus and better corrosion resistance compared to the commercially pure titanium or the most popular Ti-6Al-4V alloy. 

Alloys with additions of Zr, Nb and Ta show good biocompatibility and these elements belong to the vital group in the tissue reaction (they belong to the non-toxic and non-allergic group of elements) [2,3,4,5,6]. The titanium alloys composed of such elements have been intensely developed for more than a decade [2,3,4,5,6,7,8,9]. There are many papers reporting on binary Ti-Ta [10,11,12,13,14,15,16] alloys. Tantalum has excellent corrosion resistance and appears to be a good alloying element to the titanium alloys. Ti-Ta binary alloys have recently attracted interest due to a good combination of high strength and low modulus [10]. For example, Ti-30Ta alloy has an elastic modulus of 77 GPa and the tensile strength of 713MPa [10].

One of the new methods of preparation of porous metals developed by our group is thermal dealloying of Mg. In the previous paper, we described the formation of a metallic foam by removing Mg during the sintering process and the development of a scaffold based on the tantalum-based alloys and composites [16,17]. In the current paper, we focus on the preparation and characteristics of Ti-Ta β alloys with a high content of magnesium. The alloys developed in this work will be used as precursors for the preparation of metallic foams/scaffolds. Such structures are characterized by high porosity of up to 70%. In order to achieve that in the process of thermal dealloying of magnesium, a relatively high Mg content in the precursor alloy is necessary. In this study, the authors prepared ternary alloys based on Ti-30Ta with 30–50 wt% of magnesium.

## 2. Materials and Methods 

### 2.1. Materials Preparation

The nanostructured ternary alloys were prepared by mechanical alloying (MA) in an Spex SPEX 8000 Mixer Mill filled with argon gas. The ball to powder weight ratio (B/P) was 20:1. The composition of alloys was as follows: Ti-30Ta-(30 ÷ 50)Mg. The initial powders were: Ti – 325 mesh, purity 99.5%, Ta – 100 mesh, purity 99.98%, and Mg – 325 mesh, purity 99.8% (all from AlfaAesar). The process batch yield was defined as the ratio of the weight of the recovered powder and the weight of the starting powder measured using a precision balance (0.001 g repeatability). These measurements were performed in the glove box (LabMaster 130) filled with automatically controlled argon atmosphere (O_2_ < 2 ppm and H_2_O < 1 ppm).

The consolidation step was as follows: The powder after MA was portioned, placed into a steel die and uniaxially pressed at a pressure of 1000 MPa. The green compacts were 10 mm in diameter and 5–6 mm in height. The sintering/dealloying step was carried out in the tube furnace (Nabertherm, Lilentahl, Germany) at 1300 °C for 10.8 ks, in 10^−2^ Pa vacuum to remove the magnesium vapors from the material and prevent excessive oxidation. Then, the sinters were slowly chilled to the room temperature together with the furnace.

### 2.2. Phase and Structural Analysis

The phase constitution and the crystallographic structure of the Ti-based powders were analyzed by the X-ray powder diffraction (XRD) with Cu Kα radiation (Panalytical, Empyrean model, Almelo, Netherlands). The conditions of the XRD measurements were as follows: voltage 45 kV, anode current 40 mA, 2theta range 30°–90°, time per step 60.214 s, step size 0.0245°, λ = 1.54 Å.

The XRD data were used to calculate the average crystallite size and the lattice strain using the Williamson-Hall plot and the Panalytical HighScore 3.0a (Panalytical, Almelo, Netherlands) software. All computations, based on the XRD data, were made after subtracting the background and the peak fittings. The refinement of the diffraction data was performed using the Rietveld refinement technique with the Maud 2.91 software (by Luca Luttorotti, Univ. of Trento, Italy). The instrumental peak broadening was evaluated using an Si reference sample. The Marquardt Least Squares based on the Levenberg-Marquardt algorithm was used in order to minimize the difference between the experimental and the calculated patterns. The refinement analyses were carried out using space groups and crystallographic information from the Crystallography Open Database (β-Ti: COD 9012924).

### 2.3. Microstructure and Porosity Analysis

A scanning electron microscope (SEM, Mira 3, Tescan, Brno, Czech Republic) was applied for the investigations of the morphology and the porosity of the sinters. 

The microstructure of the obtained materials was studied by the CM 20 Super Twin (Philips, Amsterdam, Netherlands) transmission electron microscope (TEM) microscope providing 0.24 nm resolution at an acceleration voltage of 200 kV. The powders for the TEM observation were ultrasonically distributed in ethanol in the time of 60 s and one drop of such a suspension was transferred onto the EMS support film on the grids (square mesh standard thickness FF100–Cu 100 mesh).

### 2.4. Measurements of Mechanical Properties

The compressive strength and elastic moduli were measured using a 4483 Instron mechanical testing machine of the measuring range of up to 20 kN with a constant crosshead speed (the average strain rate of 0.001 s^−1^). The mechanical properties were measured on eight samples of each series. The Vickers hardness (HV) and the indentation modulus (E_IT_) were also measured at loading using the Picodentor HM500 (Fischer, Sindelfingen, Germany) nanoindentation tester. The indentations were made using a diamond indenter at the force of 300 mN for 20 s.

### 2.5. MTT Assay

Normal human osteoblast (CC-2538) (NHost) and human periodontal ligament fibroblasts (CC-7049) (HPLF) (Lonza Group Ltd., Basel, Switzerland) were used for the in vitro cytocompatibility tests. The tests were obtained under static conditions. The samples were sterilized by autoclaving at 120 °C for 15 min and separately located in 24-well microplates. The cells were cultured at a concentration of 5000 cells/well in 1 mL of culture medium on each sample at 37 °C in a 5% CO_2_ incubator for 1, 3 and 4 days. The proliferation of the cells in the conditioned mediums was expressed as a percentage of the value of relative viability of the cells (RVC) of the reference medium. The pure bulk microcrystalline titanium samples were used for preparation of the reference medium.

The statistical significance was analyzed using Kruskal-Wallis One Way Analysis of Variance on Ranks with multiply repetition option SigmaStat 3.5 (Systat Software Inc., Erkrath, Germany) with U-Mann Whitney test. The significance level was p-value < 0.05.

## 3. Results and Discussion

Mechanical alloying is a ball-milling technique used in the preparation of non-equilibrium nanomaterials (alloys and composites). In this work, the authors show the possibility of developing the β-Ti alloys containing more than 30 wt% of magnesium, which is a very high value. In all probability, it was possible to achieve using a high B/P ratio (high milling energy).

**Figure 1:**
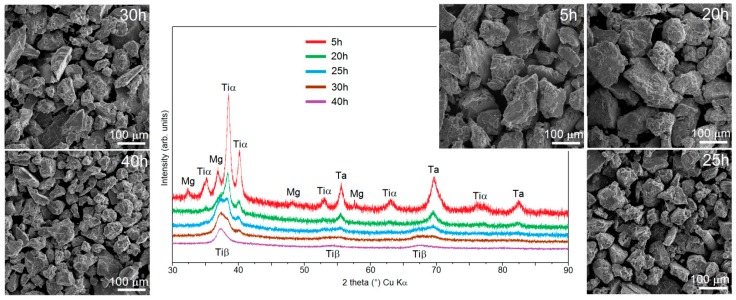
X-ray powder diffraction (XRD) spectra of the Ti-30Ta-30Mg and scanning electron microscope (SEM) micrographs of the powders at different milling times.

In this work, the preparation by mechanical alloying was shown of the β-Ti alloys, containing 30, 40 and 50 wt% of magnesium. The composition of alloys was as follows: Ti-30Ta-(30 ÷ 50)Mg wt%. The changes in the phase constitution during MA were observed using XRD and the morphology of the powders using SEM. Figure 1 shows the XRD data for the Ti-30Ta-30Mg alloy after milling. After 5 h of milling, there were well visible peaks of Ti, Ta and still visible peaks of Mg on the XRD spectra. Such a short time allowed us to achieve just a mixture of the initial powders. An increase in the milling time resulted in a decreased intensity of the XRD peaks of the initial powders and the appearance of the new XRD peaks corresponding to β-Ti. After 40 h of MA only the β-Ti peaks were observed. During the MA process, a phase transformation was observed: the peaks corresponding to the beta stabilized element (Ta) disappeared on the XRD spectra after a long milling time and the intensity of the peaks corresponding to Mg and α-Ti decreased. However, after approx. 20 h, the peaks corresponding to the β-Ti structure became visible. The intensity of these peaks increased and between the 25th and 30th hour, the intensities of the α-Ti and β-Ti peaks (between the 2 theta 35th–40th degree) became equal. After 40 h of milling for the alloy containing 30% of Mg, there were only peaks corresponding to β-Ti. 

**Figure 2:**
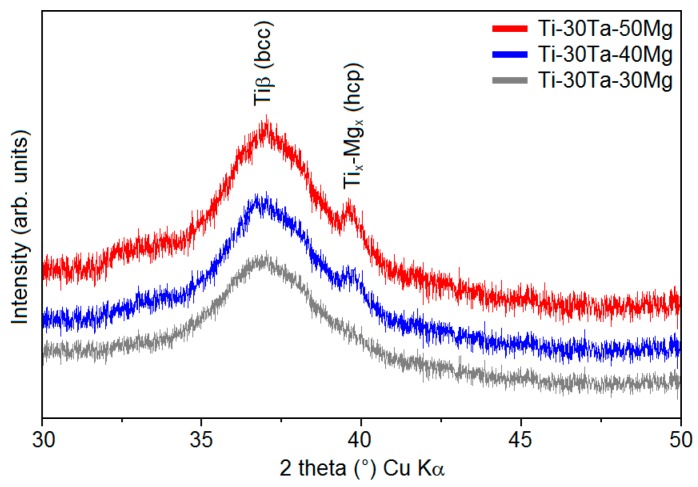
XRD spectra of the Ti-30Ta-(30-50)Mg alloys after 40 h of MA process.

When the content of Mg increased, an additional phase was visible (Figure 2). The XRD spectra for the alloy with 40 wt% and 50 wt% of Mg has been shown in Figure 2. Mechanical alloying with such a high content of Mg resulted in the appearance of an extra phase (Ti-Mg with hcp structure).

The SEM micrographs of powders at the subsequent MA stages (Figure 1) show that all of the powders are characterized by fractured and porous morphology and are composed of irregular particles/agglomerates typical of mechanically alloyed materials. After a long milling time, we can observe the following trend: with the increasing milling time, the size of the particles decreases from over 200 μm to approx. 50 μm after 5 h and 40 h of milling, respectively, which is natural to observe during MA.

One of the advantages of using Mg as an alloying element to Ti is very good yield characteristics. Zadra showed that using a small amount (0.5%) of Mg results in approx. 65% yield after 2 h of milling [18]. If we compare the results at hand with the authors’ previous work [19], it could be concluded that the yield increases with the increasing Mg content. In this work, the authors use a relatively high content of Mg. Such alloys are characterized by outstanding yield results even after a relatively long milling time. After 40 h of the MA process, more than 94% of the powder yield was achieved for all chemical compositions. 

**Figure 3:**
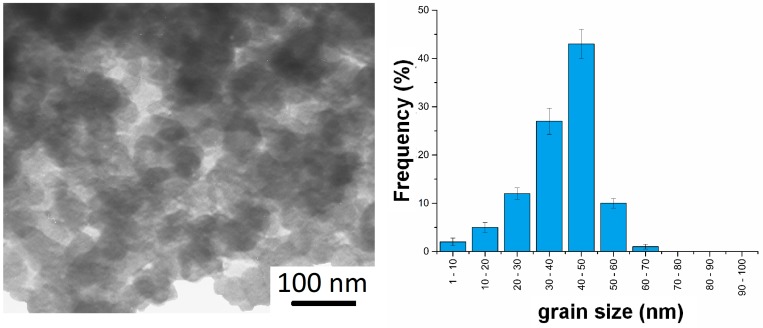
Example of TEM micrograph and the grain size distribution of the Ti-30Ta-30Mg alloy.

The microstructure was investigated by TEM. Using the mechanical alloying process, we can obtain amorphous, nanocrystalline and ultra-fine-grained materials. Figure 3 shows the example TEM micrograph and the grain size distribution of the Ti-30Mo-30Mg alloys. The results for all alloys together with the structural analysis data have been shown in Table 1. The applied algorithm achieves high goodness of fit (GOD).

In all the prepared alloys, we can observe the nanocrystalline grains. On the TEM micrographs, homogenous round-shaped nanograins are visible. Alloys containing 50% of Mg are made of crystals of larger diameter (approx. 70–90 nm) than those with 30% of Mg (approx. 30–50 nm). The fracturing could be more difficult with a higher content of Mg as a result of smaller accumulation of the lattice defects and the newly obtained Ti-Mg phase. It is obvious that the chemical composition influences the lattice parameters. An increase in the content of Mg clearly increases the cell volume, which also depends on the type of other alloying elements but in a relatively less significant way. The TEM analyses were compared with the Williamson-Hall method. The main reasons for the incompatibility could be the incompatible profile fit after the refinement of the XRD data. The most reliable technique is TEM. The results are similar for all the used techniques and the size of the crystals is confirmed. The obtained nanostructure may be helpful in the consolidation stage. The large number of grain boundaries (typical of nanomaterials) is a relatively simple way leading to the diffusion and evaporation of Mg during the high temperature dealloying process. 

**Figure 4:**
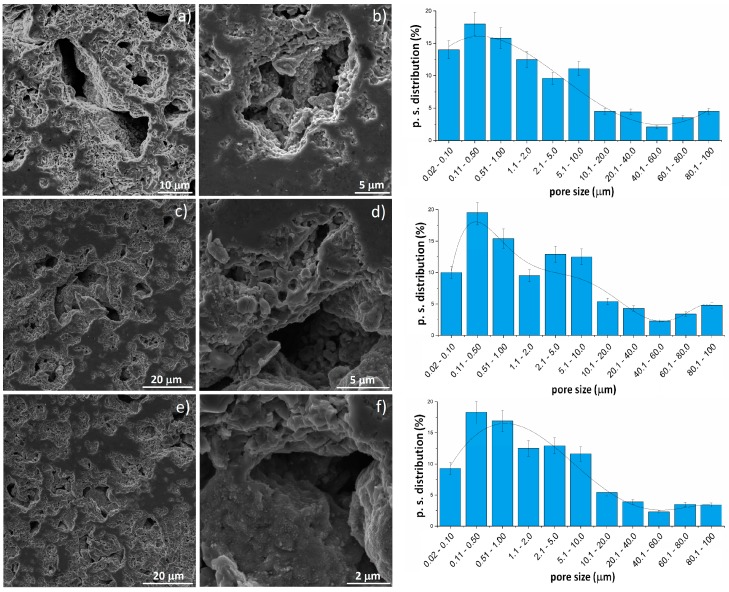
SEM image of: (**a,b**) Ti-30Ta (+initial 30 Mg), (**c,d**) Ti-30Ta (+initial 40 Mg), (**e,f**) Ti-30Ta (+initial 50 Mg) metallic foams.

The sintering stage was performed in vacuum. When the temperature rises above the Mg boiling point, the magnesium atoms start to separate from the titanium alloy structure. This moment is the start of the thermal dealloying process during which the porosity increases. When the dealloying process is underway, the magnesium atoms diffuse from the middle to the surface of the sample and combine to form vapors and then evaporate leaving open spaces surrounded by the metallic scaffold. Thermal dealloying of magnesium results in the formation of interconnected pores with a relatively wide range of pore size distribution (from nano- to micro-scale). An example of microscopic images of broken samples along with the pore size distribution have been shown in Figure 4. There are a lot of large (5 to over 90 μm) interconnected pores elongated in shape that are approx. 50% of the total pores. Another group of pores is much smaller (approx. 0.1 to 2 μm) and globular in shape, located mostly in areas between the larger pores. The total porosity is approx. 60%, 72% and 76% for the alloys with 30%, 40%, and 50% of Mg, respectively. There is a trend of porosity increase with the increasing Mg content, however, the increase is not proportional to the changes of the chemical composition. The pores are interconnected and will determine mechanical properties, which is very attractive in the biomaterial-related aspect.

The mechanical properties were measured in the compression tests and using a nanoindentation tester (Table 2). The authors chose those two techniques to measure the properties at different aspects: the compression test will provide information about the whole sample including the porosity while in the nanoindentation test, the influence of porosity is limited.

When comparing the elastic modulus results obtained with the compression test and nanoindentation test, it could be seen that the values are totally different. The modulus measured by the nanoindentation test is much higher than that obtained by the compression test. This is because the porosity level for nanoindentation is not fully taken into account, as is in the compression measurements. There is a high probability that using the nanointender we can measure the moduli appropriate for the bulk alloys. It was averagely 120 GPa for all samples, which is close to pure Ti and higher than for the Ti-30Ta alloy. However, in the investigations of porous materials, it is more important to include the influence of porosity. The moduli measured by the compression machine oscillate between 0.65 and 0.5 GPa. Those values are insignificantly higher than those measured for the Ta-Ti foams, presented in the authors’ previous work [16], which confirms the conclusion of negligibility of the impact of the chemical composition on the porosity in highly porous materials (60%–80%). The hardness of the prepared foams was approx. 300 HV, which was higher than pure titanium and comparable to some of the titanium alloys. The main factor influencing the hardness appears to be the solid solution strengthening.

**Figure 5:**
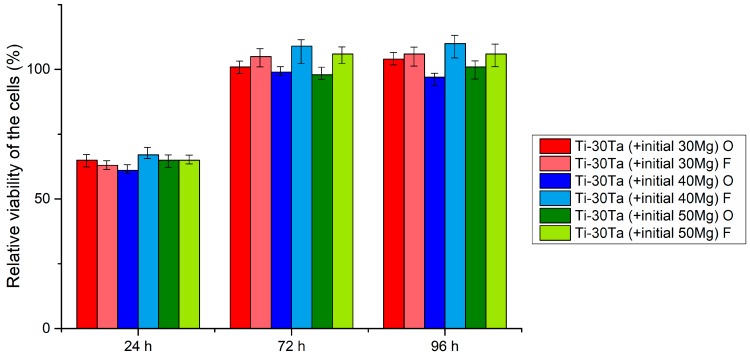
Results of the MTT assay performed at 24 h, 72 h and 96 h. (O)—NHost and (F)—HPLF cells.

Cytotoxic activity was analyzed using the MTT assay. The obtained RVC results have been shown in Figure 5. The reference medium conditioned with pure bulk microcrystalline titanium was represented by 100%. After 24 h, the RVC for all the tested materials was lower (above 60%) than that of the reference sample. The possible reason for such a situation could be the richer chemical composition of new materials than reference sample and consequent longer adaptation time of cells to the medium. However, after three and four days, the proliferation of both the osteoblasts and fibroblasts cells in all of the media conditioned with the tested porous alloys rebalanced the rate of the cell proliferation (approx. 98%–110%) in the medium conditioned with reference titanium sample. Such results allow a conclusion that all the prepared Ti based foams are non-toxic.

## 4. Conclusions

In the paper, the authors have shown a development and characteristics of the Ti beta alloys contain a high content of magnesium. These new materials were the precursors for the preparation of titanium-based metallic foams using a new thermal dealloying technique. They will find application in the production of hard tissue implants. Based on this study, the following conclusions can be drawn:

- Using a beta stabilizer such as Ta, it is possible to achieve a beta titanium structure in the presence of Mg during mechanical alloying;
- Using magnesium as the alloying element to titanium alloys, cold welding utilizing a shaker-type mill and a high B/P ratio does not appear to be an issue;
- Mechanical alloying is beneficial in the synthesis of the nanocrystalline Ti-Ta-Mg alloys;
- Thermal dealloying of magnesium results in the formation of a porous interconnected structure with wide range of the pore size (from nano- to micro-scale);
- The new foams present attractive mechanical and biological properties and may be good candidates for hard tissue implant application.

## Figures and Tables

**Table 1:** Crystallographic data of the powders after mechanical alloying.

Alloy	D [nm]	a [Å]	V [Å^3^]	Q [%]	Q _Ti-Mg hcp_ [%]	R_wp_ [%]	R_exp_ [%]	GOD
Ti-30Ta + 30Mg	44 ± 8	3.3556	37.78	100	-	4.81	2.85	1.45
Ti-30Ta + 40Mg	53 ± 6	3.3659	38.13	100	-	5.66	3.23	1.70
Ti-30Ta + 50Mg	87 ± 12	3.3778	38.54	95.92	4.08	6.58	3.49	1.98

D—crystallite size, a—lattice parameter, V—lattice volume, Q—amount of phase, R_wp_—weighted pattern residual indicator, R_exp_—expected residual indicator, GOD—goodness of fit.

**Table 2:** Mechanical properties of alloys after sintering/dealloying process.

Alloy.	Compression Test	Nanoindentation Test		
-	Compression Strength (MPa)	Elastic Modulus (GPa)	Hardness (HV)	Elastic Modulus (GPa)
Ti-30Ta (+initial 30 Mg)	14.60 ± 0.91	0.65 ± 0.03	307.08 ± 23.9	120.45 ± 8.97
Ti-30Ta (+initial 40 Mg)	10.81 ± 1.03	0.56 ± 0.03	299.60 ± 17.10	119.20 ± 8.76
Ti-30Ta (+initial 50 Mg)	9.96 ± 0.93	0.53 ± 0.03	305.05 ± 19.01	117.73 ± 7.89

## References

[1] Noort R.V. (1987). Review Titanium: The implant material of today. J. Mater. Sci..

[2] Davidson J.A., Mishra A.K., Kovacs P., Poggie R.A. (1994). New surface-hardened, low-modulus, corrosion-resistant Ti-13Nb-13Zr alloy for total hip arthroplasty. Biomed. Mater. Eng..

[3] Lugowski S.J., Smith D.C., McHugh A.D., Loon V.J. (1991). Release of metal ions from dental implant materials in vivo: Determination of Al, Co, Cr, Mo, Ni, V, and Ti in organ tissue. Biomed. Mater. Res..

[4] Okazaki Y., Rao S., Ito Y., Tateishi T. (1998). Corrosion resistance, mechanical properties, corrosion fatigue strength and cytocompatibility of new Ti alloys without Al and V. Biomaterials.

[5] Niinomi M., Hattori T., Morikawa K., Kasuga T., Suzuki A., Fukui H., Niwa S. (2002). Development of low Rigidity β-type titanium alloy for biomedical applications. Mater. Trans..

[6] Niinomi M., Nakai M., Hieda J. (2012). Development of new metallic alloys for biomedical applications. Acta Biomater..

[7] Zhang L.B., Wang K.Z., Xu L.J., Xiao S.L., Chen Y.Y. (2015). Effect of Nb addition on microstructure, mechanical properties and castability of β-type Ti-Mo alloys. T. Nonferr. Metal. Soc..

[8] Cardoso F.F., Ferrandini P.L., Lopes E.S.N., Cremasco A., Caram R. (2014). Ti-Mo alloys employed as biomaterials: Effects of composition and aging heat treatment on microstructure and mechanical behavior. J. Mech. Behav. Biomed..

[9] Zhang W.D., Liu Y., Wu H., Song M., Zhang T.Y., Lan X.D., Yao T.H. (2015). Elastic modulus of phases in Ti-Mo alloys. Mater. Charact..

[10] Zhou Y.L., Niinomi M. (2009). Ti-25Ta alloy with the best mechanical compatibility in Ti-Ta alloys for biomedical applications. Mater. Sci. Eng. C.

[11] Liu Y., Li K., Wu H., Song M., Wang W., Li N., Tang H. (2015). Synthesis of Ti-Ta alloys with dual structure by incomplete diffusion between elemental powders. J. Mech. Behav. Biomed..

[12] Mareci D., Chelariu R., Gordin D.M., Ungureanu G., Gloriant T. (2009). Comparative corrosion study of Ti-Ta alloys for dental applications. Acta Biomater..

[13] Morgado T.L.M., Navas H., Brites R. (2016). Wear study of Innovative Ti-Ta alloys. Procedia Struct. Integrity.

[14] Zhou Y.L., Niinomi M., Akahori T. (2004). Effects of Ta content on Young’s modulus and tensile properties of binary Ti-Ta alloys for biomedical applications. Mater. Sci. Eng. A.

[15] Sing S.L., Yeong W.Y., Wiria F.E. (2016). Selective laser melting of titanium alloy with 50 wt% tantalum: Microstructure and mechanical properties. J. Alloy. Compd..

[16] Adamek G. (2017). Tantalum foams prepared by the thermal dealloying process. Int. J. Refract. Met. H..

[17] Adamek G. (2019). Tantalum-45S5Bioglass composite foams prepared in thermal dealloying process. Int. J. Refract. Met. H..

[18] Zadra M. (2013). Mechanical alloying of titanium. Mater. Sci. Eng. A.

[19] Adamek G. (2014). Mechanical Alloying of Ti-20Ta-20Nb-(10 ÷ 20)Mg Alloys. Acta Phys. Pol. A.

